# Mutator Mutations Enhance Tumorigenic Efficiency across Fitness Landscapes

**DOI:** 10.1371/journal.pone.0005860

**Published:** 2009-06-10

**Authors:** Robert A. Beckman

**Affiliations:** Simons Center for Systems Biology, Institute for Advanced Study, Princeton, New Jersey, United States of America; University of Stellenbosch, South Africa

## Abstract

**Background:**

Tumorigenesis requires multiple genetic changes. Mutator mutations are mutations that increase genomic instability, and according to the mutator hypothesis, accelerate tumorigenesis by facilitating oncogenic mutations. Alternatively, repeated lineage selection and expansion without increased mutation frequency may explain observed cancer incidence. Mutator lineages also risk increased deleterious mutations, leading to extinction, thus providing another counterargument to the mutator hypothesis. Both selection and extinction involve changes in lineage fitness, which may be represented as “trajectories” through a “fitness landscape” defined by genetics and environment.

**Methodology/Principal Findings:**

Here I systematically analyze the relative efficiency of tumorigenesis with and without mutator mutations by evaluating archetypal fitness trajectories using deterministic and stochastic mathematical models. I hypothesize that tumorigenic mechanisms occur clinically in proportion to their relative efficiency. This work quantifies the relative importance of mutator pathways as a function of experimentally measurable parameters, demonstrating that mutator pathways generally enhance efficiency of tumorigenesis. An optimal mutation rate for tumor evolution is derived, and shown to differ from that for species evolution.

**Conclusions/Significance:**

The models address the major counterarguments to the mutator hypothesis, confirming that mutator mechanisms are generally more efficient routes to tumorigenesis than non-mutator mechanisms. Mutator mutations are more likely to occur early, and to occur when more oncogenic mutations are required to create a tumor. Mutator mutations likely occur in a minority of premalignant lesions, but these mutator premalignant lesions are disproportionately likely to develop into malignant tumors. Tumor heterogeneity due to mutator mutations may contribute to therapeutic resistance, and the degree of heterogeneity of tumors may need to be considered when therapeutic strategies are devised. The model explains and predicts important biological observations in bacterial and mouse systems, as well as clinical observations.

## Introduction

Tumorigenesis is a multistep process [1]–[5], likely including genetic mutations in oncogenes and tumor suppressor genes.

The mutator hypothesis states that mutations leading to enhanced genomic instability (termed “mutator mutations”) drive cancer pathogenesis by accelerating the acquisition of oncogenic mutations. Originally formulated around DNA polymerases and repair enzymes [6], the mutator hypothesis has been broadened to include microsatellite instability, chromosomal instability, and deficits in checkpoint activation [7]–[10]. Although mutator mutations have been found in the germline in certain familial cancer syndromes [7]–[8], the generalized mutator hypothesis focuses on *somatic* mutator mutations occurring as a step in the evolution of somatic cells towards malignancy.

On the contrary, it has been argued that mutator mutations (MM) are unnecessary for cancer development, and that the observed incidence rates of cancer may be explained by mutations occurring at the normal rate in conjunction with multiple rounds of lineage expansion and selection [11]–[14]. The debate concerning the relevance of the mutator hypothesis has centered around whether mutator mechanisms are required to explain the appearance of a single cancer cell within a human lifetime.

A novel approach was recently suggested, based on the wider perspective that all potential mechanisms of tumorigenesis are in play, but those which produce malignant lineages most efficiently are most likely to contribute to clinical cancers [15]. *Efficiency* is defined as the expected number of malignant lineages generated up to and including a reference timepoint by any particular tumorigenic mechanism. This shifts the issue from analyzing the waiting time to a single cancer cell, and fitting it to epidemiologic data, to the evaluation of the *relative efficiencies of mutator and non-mutator pathways in cancer lineage production*.

In this framework, mutator mutations, lineage expansion, and selection are not mutually exclusive and could all simultaneously contribute to tumorigenesis. It is also noted that the conversion rate of normal cells to cancer cells (“cancer lineage birth rate”) likely far outstrips the number of clinically observed cancers, due to numerous malignant and premalignant lineages being eliminated by immune surveillance, failure to establish a blood supply, or competition from other premalignant lineages. Thus models which match the cancer lineage birth rate to clinical cancer incidence may have inherent limitations. It may be more relevant to evaluate the potential contribution of mutator mutations to the efficiency of tumorigenesis, as opposed to whether mutator mutations are necessary to explain a rate of cancer lineage birth rate equal to that of clinically observed cancer incidence. Furthermore, attempts to compare absolute theoretical cancer rates to absolute observed cancer rates are very sensitive to the underlying parameters and other assumptions, leading to variability in conclusions [16], whereas in the calculation of relative efficiencies, many parameters cancel in the ratios, minimizing the danger of overfitting of models and providing the potential for more robust conclusions. It is assumed in these models that any given malignant lineage has an approximately constant and low probability of developing into a clinical cancer.

An analysis of the relative efficiency of tumorigenesis with and without a somatic mutator mutation, in the absence of lineage expansion (LE), demonstrated that mutator mutations enhance tumorigenic efficiency under many realistic scenarios, despite the need for an extra mutation step to acquire the mutator mutation itself [15]. Mutator mutations generally do not enhance efficiency for cancers whose pathogenesis requires only two genetic alterations, but increase dramatically in importance as the number of steps in tumorigenesis increases. However, as the model did not explicitly include lineage selection and expansion, the question of the contribution of mutator mutations to tumorigenic efficiency in the presence of lineage selection and expansion remained open.

Mutator lineages are also more likely to suffer deleterious mutations that reduce their fitness and potentially lead to extinction. This effect has been termed negative clonal selection (NCS) [17]. To date, no analysis of this effect integrated with simultaneous genetic evolution of the tumor has been performed.

In order to account for the effects of selection and expansion of fitter lineages, as well as negative clonal selection, I systematically consider the *fitness landscape*, or the multidimensional space representing cellular fitness, as a function of cellular genetic makeup within an environmental context. Pathways through this fitness landscape are termed *fitness trajectories*. Trajectories of special interest for tumorigenesis are those which begin with a normal cell and end with a transformed malignant cell.

**Figure 1:**
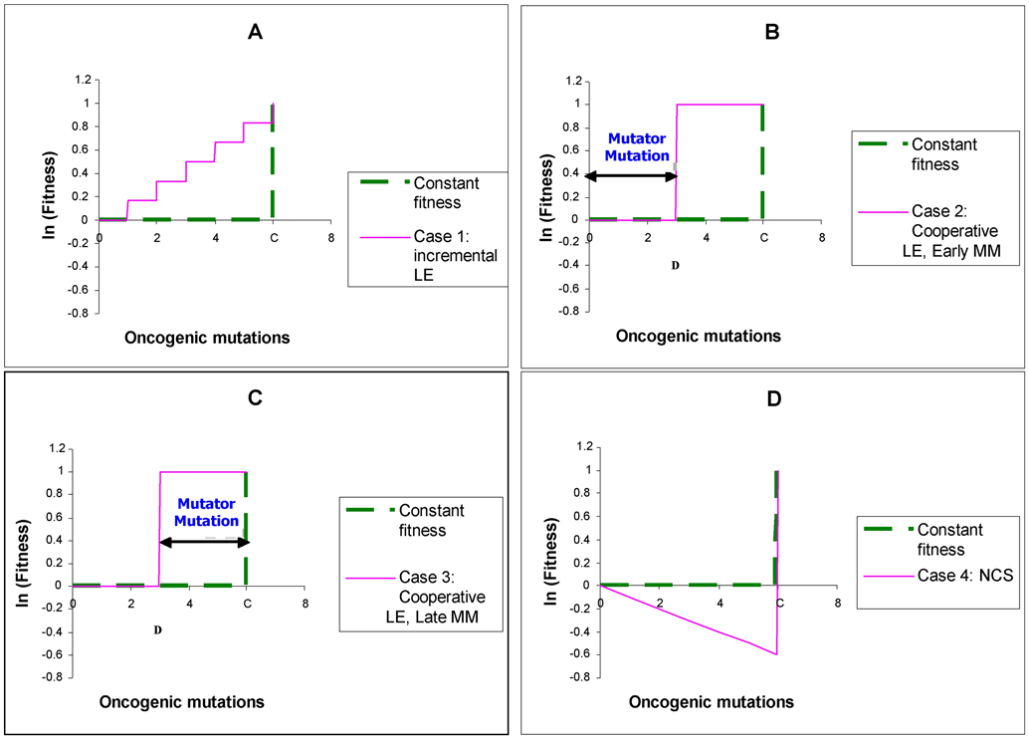
Representative fitness landscapes for tumorigenesis. R, the natural logarithm of the relative fitness advantage compared to wild type, is plotted as a function of number of oncogenic mutations for each of the four fitness landscapes considered in this paper (pink lines), relative to the constant fitness case (green lines) [15]. Positive and negative values of R correspond to increased and decreased fitness respectively. In this figure, it is assumed that C oncogenic mutations are required for malignant transformation, at which point the lineage acquires markedly increased fitness relative to wild type. A, Case 1: incremental lineage expansion (LE). The relative fitness increases incrementally with each oncogenic mutation. B, Case 2: cooperative lineage expansion with early mutator mutation (MM). Fitness increases suddenly and cooperatively after a predefined number, D<C, of oncogenic mutations, prior to malignant transformation after C mutations. In the mutator pathway, the mutator mutation occurs *before* the sudden increase in fitness, within the time bounded by the arrows. C, Case 3: cooperative lineage expansion (LE) with late mutator mutation (MM). As in B, except in the mutator pathway the mutator mutation occurs *after* the sudden fitness increase, within the time bounded by the arrows. D, Case 4: negative clonal selection (NCS). The lineage acquires oncogenic mutations, while the fitness continuously decreases due to accumulated random deleterious mutations. The fitness of the lineage increases only if it reaches full malignant transformation.

This paper presents mathematical models which represent the general case of tumorigenesis across a variety of fitness trajectories, including multiple situations where the mutator lineage suffers reduced fitness (NCS), or achieves increased fitness leading to lineage expansion (LE). Four cases (“fitness trajectories”), which differ in the fitness of intermediate lineages in the tumorigenic process (Figure 1), are considered for both mutator and non-mutator pathways with respect to the production or birth rate of new malignant lineages. In order to become a malignant lineage, a normal lineage must accumulate a fixed number of oncogenic mutations (hits). Lineages with less than the full complement of oncogenic mutations may still expand their relative numbers, or risk extinction, according to their relative fitness, on a continuous basis throughout the process. The cases differ with respect to the assumed fitness trajectory, i.e. the relative change in fitness with each successive oncogenic mutation (Figure 1).

In case 1, the *incremental* lineage expansion case, the lineages acquire a fixed increment in fitness with each successive oncogenic mutation, finally achieving their maximum fitness when they have acquired a full complement of oncogenic mutations. Given that most of the increased tumorigenic efficiency due to a mutator mutation can be captured by the case in which the mutator mutation is an initial step [15], we focus in the mutator pathway analysis for case 1 on mutator mutations occurring as an initial step. In case 2, the *cooperative* lineage expansion case with *early* mutator mutation, there is no increase in fitness until a subset of oncogenic mutations have been acquired, at which point the fitness increases rapidly and lineage expansion begins. Additional oncogenic mutations may then be required to achieve the fully malignant phenotype. The mutator mutation occurs early, e.g. at any point before the lineage expansion. In case 3, the *cooperative* lineage expansion case with *late* mutator mutation, the situation is analogous to case 2 except that the mutator mutation occurs after the onset of lineage expansion, during the period when additional oncogenic mutations are occurring towards reaching a fully malignant phenotype. Since cases 2 and 3 are alternate mutually exclusive subsets of the same fitness trajectory, their relative efficiencies (compared to non-mutator pathways) are additive. In case 4, the mutator and wild type lineages are subject to negative clonal selection [17]. The lineages have a subset of loci (reduced fitness or “RF” loci), mutation of which *may* lead to reduction in fitness, depending on the genetic and environmental context. When a reduced fitness locus is mutated, the lineage is at risk for fitness reduction. Lineages with fitness reduction become extinct, thus potentially limiting the advantage conferred by a mutator mutation.

While cases can be proposed that are mixtures of these four cases, it should be possible to infer their properties once these four archetypal fitness trajectories are analyzed. Thus, based on analysis of these pathways in combination (together with the constant fitness pathway previously analyzed [15]), any conceivable fitness landscape could be analyzed.

Using these models, I evaluate the relative contribution of mutator mechanisms to tumorigenesis, considering in a quantitative fashion those issues which have historically been raised as counterarguments to the mutator hypothesis, and demonstrating predominance of mutator pathways in most instances.

In addition, in the presence of negative clonal selection, I find an optimal mutation rate for tumor evolution, which appears to differ from that for species evolution.

The models are focused on enhanced single base substitution rates, and it would be of interest to specifically model other forms of genetic instability that might lead to deletions or to chromosomal instability.

The analysis raises several provocative questions:

1. As mutator pathways appear to predominate in most instances, can the diversity and complexity of tumors be addressed by current therapeutic strategies?
2. Can tumor diversity and genetic instability be used to stratify patients for prognosis and therapy?
3. Can therapy be designed to increase the mutation rate in tumors beyond the optimum derived in this paper, resulting in lethal mutagenesis?
4. Can the onset of tumors be delayed to beyond the human lifetime, and therefore prevented, by small decreases in the mutation rate?
5. What are the underlying reasons for quantitative differences between tumor and species evolution?

## Results

### Model outputs

The results for the four cases below are presented in terms of two key model outputs: (1) relative tumorigenic efficiency of mutator vs. non-mutator pathways, N_rel_, and (2) the minimum fold increase in mutation rate required from a mutator mutation before the mutator pathway has a relative tumorigenic efficiency greater than or equal to 1, termed α_50%_.

N_rel_ is the ratio of malignant lineages produced by mutator and non-mutator pathways under the specified conditions. The fraction of clinical cancers arising by mutator pathways is given by N_rel_/(1+N_rel_), and mutator mechanisms predominate if N_rel_>1. In non-lineage expansion models, in which progression to a malignant lineage is a rare event, N_rel_ can be expressed as a ratio of probabilities, P_rel_ [15].

α is the multiplicative factor by which a mutator mutation increases the somatic mutation rate per cell generation, e.g. the magnitude of the genetic instability. In the lineage expansion (cases 1–3) and constant fitness [15] models, there is a minimum value of α, which we term α_50%_, at which mutator pathways are expected to contribute to half of clinical cancers, and above which mutator pathways predominate. The fraction of total cancers caused by a mutator pathway with a given α when compared to an alternative non-mutator pathway is given by α^C^/(α^C^+α_50%_^C^), where C is the number of oncogenic mutations required for malignant transformation. Thus, a mutator mutation must confer a minimum level of genetic instability to be relevant. In evaluating the importance of mutator pathways in a particular model, we need to determine if α_50%_ is within a range commonly seen in known mutator mutations.

Mutations in base selection and proofreading generally increase mutation rates 10–100 fold [18]–[19], and increased random mutation frequencies of up to 500-fold have recently been observed in human tumors [20]. In evaluating the results below, mutator pathways are expected to predominate when α_50%_ is at or below commonly observed values of α (ca. 10–500).

For the negative clonal selection model (case 4), an additional key output is an optimal value of the fold increase in mutation rate, α_optimal_, which maximizes the importance of mutator pathways. This corresponds to an optimal mutation rate, k_mut-optimal_.

### Model inputs

The results depend on the properties of the tumor under consideration, which in turn define the inputs to the models. The key input parameters for all the models are: C, the number of oncogenic mutations required for transformation to the malignant phenotype; R≥0, the natural logarithm of the relative fitness of a malignant cell compared to wild type (meaning that with each successive generation the relative numbers of the malignant lineage increase by a factor e^R^); 0≤R_p_≤R, the component of R which is due to enhanced proliferation (the remainder would be due to decreased apoptosis); T, the time (in cell generations) to malignancy; N_ML_, the number of genomic “mutator loci”, in nucleotides, mutation of which leads to genetic instability; and k_mut_, the mutation rate per nucleotide base per cell generation in wild type cells.

In the *cooperative lineage expansion* models (cases 2 and 3), an additional input parameter is introduced: D, the number of oncogenic mutations required for an increase in fitness. In the *negative clonal selection* model (case 4), I introduce the input parameter N_RFLN-D_ (N_reduced fitness loci net-dominant_), which is an indicator of the vulnerability of the genome to mutations which may reduce cellular fitness [17]. It consists of the number of loci, in base pairs, single copy mutation of which may reduce fitness of the lineage, where the loci are divided into subclasses, and the number in each subclass is multiplied by the probability that a mutation of it will lead to a fitness reduction as a function of genetic and environmental context.

Key input parameters and the ranges over which they have been varied in the calculations, as well as key model outputs, are summarized in Table 1. The remainder of this section describes selected results and their dependence on input parameters. Further detailed results, not shown in the Figures, are given in Supplementary Tables.

**Table 1:** Input and output parameters for models.

Parameter	Definition	Applicability	Range	References^3^
N_OL_	Number of oncogenic loci^1^	Input, all models	100	15
C	Number of oncogenic mutations required for malignant transformation	Input, all models	2–12	15, 22
D	Number of oncogenic mutations required for cooperative fitness increase	Input, cooperative lineage expansion models (cases 2 and 3)	1–6	NA
k_mut_	Wild type mutation rate^2^	Input, all models	10^−11^–10^−9^	15,17
T	Number of cell generations to cancer	Input, all models	170–5000	15,17
N_ML_	Number of loci available for mutator mutations^1^	Input, all models	100–1000	15
α	Fold increase in mutation rate due to mutator mutation	Input, all models	1–∞ (commonly 10–500)	15, 18, 22
R	Log of relative fitness advantage for malignant cells	Input, lineage expansion models (cases 1–3)	0–2	NA
R_P_	Log of relative fitness advantage due to proliferation	Input, lineage expansion models (cases 1–3)	0–1.31	Tables S1–S2
N_RFLN-D_	Indicator of vulnerability of genome to dominant reduced fitness mutations	Input, negative clonal selection model (case 4)	0–9.8×10^5^	17
N_rel_	Relative efficiency of mutator pathways compared to non-mutator pathways	Output, all models	NA	NA
P_rel_	Relative probability of mutator pathways compared to non-mutator pathways	Output, constant fitness model and negative clonal selection model (case 4)	NA	15
α_50%_	Minimal fold increase in mutation rate corresponding to at least equal efficiency of mutator and non-mutator pathways	Output, constant fitness and lineage expansion models (cases 1–3)	NA	15
k_mut-optimal_	Optimal mutation rate for tumor evolution	Output, negative clonal selection model (case 4)	NA	NA

1 in nucleotide bases.

2 per nucleotide base, per wild type cell generation.

3 references where parameter is explained or its reference range is justified; NA, not applicable.

In the calculations, I assume N_ML_ is 100, a very conservative assumption [15], [21]. If N_ML_ = 1000, α_50%_ would decrease by a factor of 10^1/C^ (relative to the same case with N_ML_ = 100), further enhancing the potential role of mutator pathways.

Using the equations in Methods, one may rapidly explore a wide variety of other questions and input parameters.

### Case 1: incremental lineage expansion

**Figure 2:**
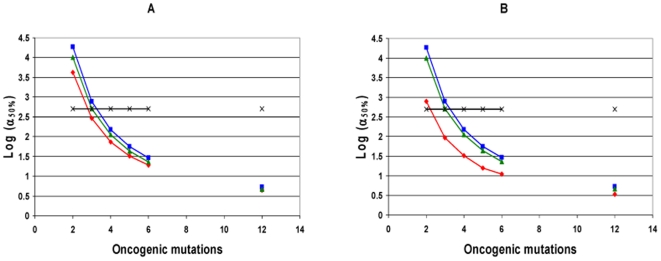
Log(α_50%_), for constant fitness and incremental lineage expansion models. The log of α_50%_, the minimum fold increase in mutation rate due to a mutator mutation at which mutator pathways contribute to 50% of cancers, plotted as a function of C, the number of oncogenic mutations required for transformation, for the constant fitness model (red), and the incremental lineage expansion model, with fitness advantage e^R^ = 1.4 (green) or 2.0 (blue). The fitness advantage due to enhanced proliferation e^RP^ = 1.2 (green) or 1.4 (blue). The black horizontal line represents α = 500. Mutator mutations with α≤500 are within the range experimentally demonstrated. All points below the black line represent scenarios where mutator pathways are favored. Results are shown at T = 170 cell generations (A) and at T = 5000 cell generations (B). Mutator pathways are favored in most instances, progressively more so as the number of oncogenic mutations required for malignant transformation increases. Incremental lineage expansion decreases the degree to which mutator pathways are favored, but only slightly, and this effect progressively lessens as the number of required oncogenic mutations increases. When 3 oncogenic mutations are required for malignant transformation, mutator pathways are favored at constant fitness, but not for incremental lineage expansion with a large fitness advantage. Comparing A and B, constant fitness mutator pathways are more favored with a larger number of cell generations T, whereas this is not the case in the incremental lineage expansion model. Calculated as in reference [15] for the constant fitness case, and using equations [12a–h] for the incremental lineage expansion cases, with wild type mutation rate k_mut_ = 10^−11^; and number of loci, mutation of which leads to a mutator mutation, N_ML_ = 100. These values are conservative, and higher values would further increase the influence of mutator pathways.

In analyzing this case, we assume that C oncogenic mutations are required for transformation to the malignant phenotype (C generally varying between 2 and 12 based on epidemiologic data of cancer incidence as a function of age [22]), that a malignant cell has increased fitness R relative to wild type (meaning that with each successive generation the relative numbers of the malignant lineage increase by a factor e^R^), and each successive oncogenic mutation leads to an incremental increase in fitness R/C (Fig. 1A). Based on the previous finding that the major component of efficiency in mutator pathways is due to initial mutator mutations [15], we evaluate the mutator pathway assuming the mutator mutation occurs first. We find in this case that multiple lineages are simultaneously expanding at different exponential rates, corresponding to lineages with 1, 2, … C−1 oncogenic mutations, and therefore incrementally different fitness. Thus the full expression for the number of malignant cells generated by either pathway is the sum of exponentials. If we approximate these expressions by the highest order term (i.e. the most rapidly growing exponential), representing the pool of cells with C−1 oncogenic mutations from which the new malignant lineages are drawn by one more mutation, we obtain several results (Supplementary Tables S1 and S2 and Figure 2). α_50%_ is calculated using equations[11–12], and N_rel_ by equation [13], in Methods.

Firstly, mutator mechanisms predominate in most instances, although the value of α_50%_ increases slightly compared to the constant fitness case (Supplementary Table S1 and Figure 2). For most parameter values in case 1, α_50%_ remains within the range of α for commonly observed mutator mutations, indicating that mutator pathways will have a significant role in tumorigenesis.

The relative importance of mutator pathways in tumorigenesis increases as the number, C, of oncogenic mutations required to generate a malignant phenotype increases, as judged by α_50%_ values. When 2 or fewer oncogenic mutations are required, non-mutator pathways predominate. When 4 or more oncogenic mutations are required, mutator pathways predominate. When 3 oncogenic mutations are required, the results depend on the parameter values (Supplementary Table S1 and Figure 2). Mutation of both copies of a recessive oncogene would count as 2 oncogenic mutations.

When compared to the constant fitness case, incremental lineage expansion limits the importance of mutator pathways when two oncogenic mutations are required for cancer (C = 2) and to some extent at C = 3 with a low wild type mutation rate (k_mut_), but for higher values of C, α_50%_ continues to be well within commonly observed ranges. For example, when three oncogenic mutations are required for cancer (C = 3), the wild type mutation rate is low (k_mut_ = 10^−11^), and the relative fitness advantage e^R^ of malignant cells relative to wild type is 2, a 770-fold increase in the mutation rate would be required for mutator pathways to be observed in 50% of the cancers (Supplementary Table S1). This increase is at or beyond the upper range of increase in mutation rate due to common mutator mutations. In contrast, when six oncogenic mutations are required for cancer (C = 6), α_50%_ ranges from 11–30 (Supplementary Table S1), in the lower range of commonly observed values of mutation rate increase due to mutator mutations, suggesting a predominance of mutator pathways, in that most mutator mutations would then correspond to α>α_50%_. Note in Supplementary Table S1 that this result for C = 6 is unchanged for all combinations of wild type mutation rate, cell generations to cancer, and degree of fitness increase within the explored parameter values.

When judged by relative efficiency N_rel 1∶0_, the importance of mutator pathways is reduced relative to the constant fitness case to a greater degree than one would judge based on α_50%_. This is because the value of N_rel 1∶0_ is very sensitive to small changes in α, and therefore a relatively small increase in α is required to compensate for the effect of incremental lineage expansion on N_rel 1∶0_. Based on the analytical model, the relative efficiency N_rel 1∶0_ is reduced in the case of incremental lineage expansion by a factor of RT (C−1)/[(C+1)C] compared to the constant fitness case. However, α_50%_ would need to increase by a factor of only {RT (C−1)/[(C+1)C]}^1/C^ to compensate for this. For example, with the relative fitness of malignant cells e^R^ = 2, the number of cell generations T = 5000, and the number of required oncogenic mutations C = 6, the relative tumorigenic efficiency N_rel 1∶0_ is reduced over 400-fold relative to the constant fitness case. But only a 2.7 fold increase in α can restore the same relative importance of mutator pathways under these circumstances.

The analytical model (equations[11–13] in Methods) shows that the relative importance of mutator pathways N_rel 1∶0_ increases with increasing wild type mutation rate k_mut_ and increasing fold-increase in mutation rate, α, similar to the constant fitness case [15].

Very large relative fitness advantages for malignant cells e^R^ somewhat further reduce the importance of mutator pathways (Figure 2, Supplementary Tables S1 and S2). For example, when three oncogenic mutations are required for cancer (C = 3), the number of cell generations to cancer T = 5,000, and the wild type mutation rate is k_mut_ = 10^−11^, α_50%_ is 510 when the relative fitness advantage e^R^ of malignant cells relative to wild type is 1.2, 770 when the relative fitness advantage is 2, and 1100 when the relative advantage is 7.4 (Supplementary Tables S1 and S2).

Finally, the analytical model (equations[11] and [13] in Methods) shows that the relative importance of mutator pathways N_rel 1∶0_ is approximately independent of the number of cell generations T, in contrast to the constant fitness case, where it is proportional to T [15]. Different cancer types are thought to typically arise after different numbers of cell generations T. The relative importance of mutator pathways in these different cancer types may thus depend on the fitness landscapes experienced by cells with less than the full complement of oncogenic mutations.

### Case 2: cooperative lineage expansion, early mutator mutation

In this circumstance, D oncogenic mutations occur leading to a sudden cooperative increase in fitness. At some time during the acquisition of these initial D mutations, a somatic mutator mutation may occur. After the acquisition of the first D oncogenic mutations, and consequent increase in fitness, an additional C–D oncogenic mutations must occur to complete the transformation to a malignant lineage (Figure 1B). α_50%_ is calculated using equation [16], and N_rel_ by equation [13], in Methods.

**Figure 3:**
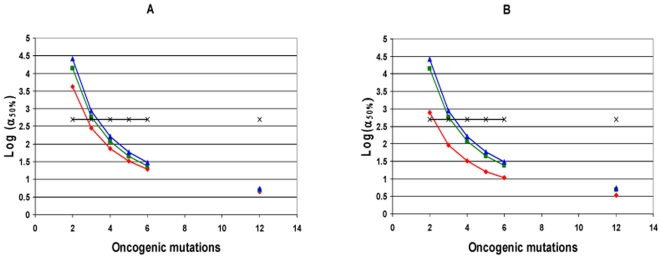
Log(α_50%_), constant fitness and cooperative lineage expansion models with early mutator mutation. The log of α_50%_, the minimum fold increase in mutation rate due to a mutator mutation at which mutator pathways contribute to 50% of cancers, plotted as a function of C, the number of oncogenic mutations required for transformation, for the constant fitness model (red), and the cooperative lineage expansion model with early mutator mutation (CLE-EMM), with fitness advantage e^R^ = 1.4 (green) or 2.0 (blue), fitness advantage due to enhanced proliferation e^RP^ = 1.2 (green) or 1.4 (blue), T = 170 cell generations (A) or T = 5000 cell generations (B). The black horizontal line represents α = 500. Mutator mutations with α≤500 are within the range experimentally demonstrated. Points below the black line represent scenarios where mutator pathways are favored. Mutator pathways are generally favored, progressively more so as the number of oncogenic mutations required for malignant transformation increases. CLE-EMM decreases the degree to which mutator pathways are favored, but only slightly, and this effect progressively lessens as the number of required oncogenic mutations increases. When 3 oncogenic mutations are required for malignant transformation, mutator pathways are favored at constant fitness, but not for CLE-EMM and a large fitness advantage. Comparing A and B, mutator pathways are more favored with more cell generations T, for the constant fitness model, but not for CLE-EMM. Calculated as in reference [15] for the constant fitness case, and using equation [16] for CLE-EMM, with the number of oncogenic mutations required for cooperative fitness increase D = 2 (except for when the number of oncogenic mutations required for malignant transformation C = 2, then D = 1); wild type mutation rate k_mut_ = 10^−11^; and number of loci, mutation of which leads to a mutator mutation N_ML_ = 100. These values are conservative, and higher values would further increase the influence of mutator pathways.

In this case also, mutator mechanisms predominate. The results with regard to α_50%_ are depicted in Figure 3. As in the incremental lineage expansion case, the calculations show a slight increase in α_50%_ relative to the constant fitness case, while still generally indicating a predominance of mutator pathways. When judged by relative efficiency N_rel_, the importance of mutator pathways is again reduced relative to the constant fitness case to a greater degree than one would judge based on α_50%_, again due to the high sensitivity of N_rel_ to the value of α, but a small change in α can compensate.

When compared to the constant fitness case, cooperative lineage expansion with early mutator mutation limits the importance of mutator pathways when few oncogenic mutations are required for cancer (C = 2) and to some extent at C = 3 with a low wild type mutation rate (k_mut_), but for higher values of C, α_50%_ continues to be well within commonly observed ranges. For example, when three oncogenic mutations are required for cancer (C = 3), the relative fitness of malignant cells e^R^ = 2, and the wild type mutation rate is low (k_mut_ = 10^−11^), an 880-fold increase in the mutation rate would be required for mutator pathways to be observed in 50% of the cancers (see Figure 3 and Supplementary Table S1). This increase is at or beyond the upper range of increase in mutation rate due to common mutator mutations. In contrast, when six oncogenic mutations are required for cancer (C = 6), α_50%_ ranges from 11–30 (Supplementary Table S1), in the lower range of commonly observed values of mutation rate increase due to mutator mutations, suggesting a predominance of mutator pathways.

The analytic models (reference [15] and equations [13–14] and [16] in this paper) show that mutator pathways are more likely if they occur early within this window, and also for higher wild type mutation rate k_mut_, and fold change α in mutation rate due to a mutator mutation, similar to the results for the constant fitness and cooperative lineage expansion with late mutator mutation cases.

Very large relative fitness advantages for malignant cells e^R^ somewhat further reduce the importance of mutator pathways (Figure 3, Supplementary Tables S1 and S2). For example, when three oncogenic mutations are required for cancer (C = 3), the number of cell generations to cancer T = 5,000, and the wild type mutation rate is k_mut_ = 10^−11^, α_50%_ is 590 when the relative fitness advantage e^R^ of malignant cells relative to wild type is 1.2, 880 when the relative fitness advantage is 2, and 1260 when the relative advantage is 7.4 (Supplementary Tables S1 and S2).

However, in contrast to the constant fitness and cooperative lineage expansion with late mutator mutation cases, the analytic model (equations [13] and [16]) shows that the relative contribution of mutator pathways is independent of number of cell generations T. Finally, the relative contribution of mutator pathways is shown by the analytic model (equations [13] and [16]) to be independent of the number of oncogenic mutations required for an increase in fitness, D, in contrast to the cooperative lineage expansion with *late* mutator mutation case.

### Case 3: cooperative lineage expansion, late mutator mutation

In this circumstance, D oncogenic mutations occur leading to a sudden cooperative increase in fitness. After this occurs, an additional C–D oncogenic mutations must occur to complete the transformation to a malignant lineage. During this latter period, a somatic mutator mutation may occur (Figure 1C). For both mutator and non-mutator pathways, the lineages will have greater numbers of cells due to their increased fitness. However, this increased fitness is constant for both types of pathways, and remains constant during the period in which the possible occurrence of a mutator mechanism is being considered. Thus, the *ratio* N_rel_ of malignant cell lineages produced by mutator and non-mutator pathways will be nearly equivalent to the probability ratio P_rel_ previously derived for the constant fitness case [15], except that the parameter C (number of oncogenic mutations required for cancer) is now replaced by C–D (the number of oncogenic mutations required for cancer after the original fitness increase), and a factor representing more rapid acquisition of the mutator mutation due to more rapid proliferation multiplies N_rel_ (if the increased fitness includes more rapid proliferation). α_50%_ is calculated using equation [20], and N_rel_ by equation [21], in Methods.

**Figure 4:**
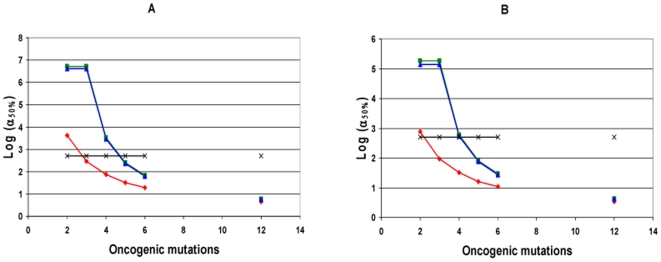
Log(α_50%_), constant fitness and cooperative lineage expansion models with late mutator mutation. The log of α_50%_, the minimum fold increase in mutation rate due to a mutator mutation at which mutator pathways contribute to 50% of cancers, plotted as a function of C, the number of oncogenic mutations required for transformation, for the constant fitness model (red), and the cooperative lineage expansion model with late mutator mutation (CLE-LMM), with fitness advantage e^R^ = 1.4 (green) or 2.0 (blue), fitness advantage due to enhanced proliferation e^RP^ = 1.2 (green) or 1.4 (blue), T = 170 cell generations (A) or 5000 cell generations (B). The black horizontal line represents α = 500. Mutator mutations with α≤500 are within the range experimentally demonstrated. Points below the black line represent scenarios where mutator pathways are favored. Mutator pathways are generally favored at constant fitness, progressively with more oncogenic mutations required for malignant transformation. CLE-LMM decreases the degree to which mutator pathways are favored, but this effect lessens with more required oncogenic mutations. When 3–4 oncogenic mutations are required for malignant transformation, mutator pathways are favored at constant fitness, but not for CLE-LMM. In contrast to other cases (Figures 2–3), a larger fitness advantage has a *small* effect in *increasing* the influence of late mutator pathways. Mutator pathways are increasingly favored with more cell generations T for all models. Calculated as in reference [15] for the constant fitness case, and using equation [20] for the cooperative lineage expansion cases, with the number of oncogenic mutations required for cooperative fitness increase D = 2 (except for when the number of oncogenic mutations required for malignant transformation C = 2, then D = 1); wild type mutation rate k_mut_ = 10^−11^; and number of loci, mutation of which leads to a mutator mutation N_ML_ = 100. These values are conservative, and higher values would further increase the influence of mutator pathways.

Mutator mechanisms predominate in most instances as long as C–D≥3 (see Figure 4 and Supplementary Tables S1 and S2), as judged by the values of α_50%_. In the case of C–D = 3, for example, α_50%_ ranges from 12 to 252, depending on various parameter values (Supplementary Tables S1 and S2). This range is well within that seen with known mutator mutations. As the number of oncogenic mutations required for cancer after the original fitness increase (C–D) increases further, greater predominance of mutator pathways is expected. For cooperative lineage expansion with C–D<3, non-mutator pathways, or mutator pathways with early mutator mutations, are more likely pathogenic mechanisms.

**Figure 5:**
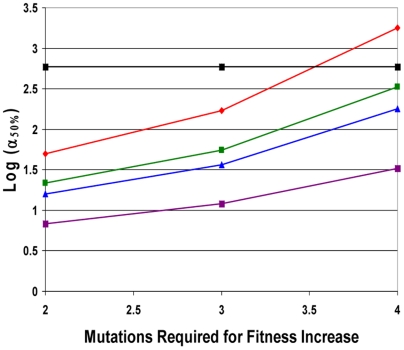
Cooperative lineage expansion: late mutator pathways less favored with increasing mutations required for fitness increase. The log of α_50%_, the minimum fold increase in mutation rate due to a mutator mutation at which mutator pathways contribute to 50% of cancers, plotted for the number of oncogenic mutations required for malignant transformation C = 6, as a function of the number of oncogenic mutations required for the cooperative fitness increase, for wild type mutation rate k_mut_ = 10^−11^ (red, green) or 10^−9^ (blue, purple), number of cell generations T = 170 (red, blue) or 5000 (green, purple), and a large fitness advantage e^R^ = 7.4 and fitness advantage due to enhanced proliferation e^RP^ = 3.7. The black horizontal line represents α = 500. Mutator mutations with α≤500 are within the range experimentally demonstrated. All points below the black line represent scenarios where mutator pathways are favored. Mutator pathways are generally favored, but late mutators (occurring after the fitness increase) are less favored the more oncogenic mutations are required for the fitness increase. Late mutator pathways are more favored in the cooperative lineage expansion case for higher wild type mutation rates k_mut_ and more cell generations to cancer T. Calculated using equation [20], with the number of loci, mutation of which leads to a mutator mutation, N_ML_ = 100. This value is conservative, and a higher value would further increase the influence of mutator pathways.

Importantly, the analytic results (equations [20–21] in Methods) imply that the importance of this pathway may depend on the number of oncogenic mutations required for increased fitness, D, when other parameters, including the number of oncogenic mutations to cancer C, are held constant. In this case, the fewer oncogenic mutations are required for increased fitness, the greater the relative predominance of this mutator pathway with late mutator mutations. The dependence of the results on D is illustrated in Figure 5 and documented for other parameter values in Supplementary Table S2. As in the constant fitness case, mutator pathways are also more likely if they occur early within this window, and for higher wild type mutation rate k_mut_, fold increase α in mutation rate due to a mutator mutation, and number of cell generations T.

For the cooperative lineage expansion case with *late* mutator mutation, the relative importance of mutator pathways is somewhat further *increased* at very large relative fitness advantages for malignant cells, e^R^ (Figure 4, Supplementary Table S2), in contrast to the incremental lineage expansion case (case 1) and the cooperative lineage expansion case with *early* mutator mutation (case 2). In the cooperative lineage expansion case with late mutator mutation, a greater fitness advantage increases the pool of cells which may acquire a late mutator mutation. For example, when the number of oncogenic mutations required for cancer C = 6, the number of oncogenic mutations required for the cooperative fitness increase D = 2, the number of cell generations to cancer T = 5,000. and the wild type mutation rate k_mut_ = 10^−11^, α_50%_ is 29 when the relative fitness advantage e^R^ of malignant cells relative to wild type is 1.2, 27 when the relative fitness advantage is 2, and 22 when the relative advantage is 7.4 (Supplementary Tables S1 and S2).

### Case 4: negative clonal selection

In this model, lineages have a constant risk per cell per cell generation of suffering a reduction in their fitness. Lineages with fitness reduction are assumed to eventually become extinct (the probability of this occurring is very high in large cell populations [17]). This phenomenon was previously studied in isolation, and termed negative clonal selection (NCS) [17]. In the current model, those lineages which do not become extinct are at the same time continuously and progressively acquiring oncogenic mutations.

The instantaneous risk of fitness reduction is the product of the mutation rate per nucleotide base per cell generation k_mut_ (or αk_mut_ after a mutator mutation) and N_RFLN-D_ (N_reduced fitness loci net-dominant_), an indicator of the vulnerability of the genome to mutation [17], consisting of the number of loci, in base pairs, single copy mutation of which may reduce fitness of the lineage, where the loci are divided into subclasses, and the number in each subclass is multiplied by the probability that a mutation of it will lead to a fitness reduction as a function of genetic and environmental context. As in the constant fitness case [15], and the incremental lineage expansion case (case 1 above), we approximate mutator pathways by considering mutator mutations occurring as an initial step in tumorigenesis. N_rel_ (which in this case is equal to the relative probability P_rel_ of mutator versus non-mutator pathways) is calculated using equations [28–30] in Methods.

In contrast to the other cases, the relative efficiency N_rel_ of mutator to non-mutator pathways does not continue to increase with greater fold increases α in the mutation rate. Increased mutation rates speed the acquisition of oncogenic mutations, but at the same time increase the risk of fitness reduction and extinction. In this type of fitness landscape, the relative efficiency N_rel_ of mutator compared to non-mutator pathways increases with greater fold increases α in mutation rate, until an optimum at which the growth of the malignant lineage begins to be limited by negative clonal selection. An approximate optimum for mutation rate k_mut_ can be estimated for this circumstance from the theoretical treatment:
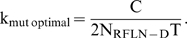
(1)


Note that the treatment focuses on dominant reduced fitness mutations only. Recessive reduced fitness mutations, requiring mutation of both alleles, were found to be quantitatively insignificant [17].

Within the parameter ranges considered within this paper, the optimal mutation rate for tumor evolution, k_mut optimal_, varies from 2.1×10^−10^ to 3.6×10^−6^ per nucleotide base per cell generation. This optimum is generally higher than estimated mutation rates of wild type embryonic stem cells or somatic cells [23]–[24], 10^−11^ to 10^−9^.

An approximately optimal value of the fold increase α in mutation rate due to a mutator mutation is therefore given by α_optimal_ = k_mut optimal_/k_mut_. In the presence of an anti-apoptotic mutation [25], the vulnerability of the genome N_RFLN-D_ would be reduced to a very low value, further raising the optimal mutation rate and diminishing the potential effect of negative clonal selection. Thus, mutator pathways with α>α_optimal_ would be more efficient if they occurred after an anti-apoptotic mutation.

**Figure 6:**
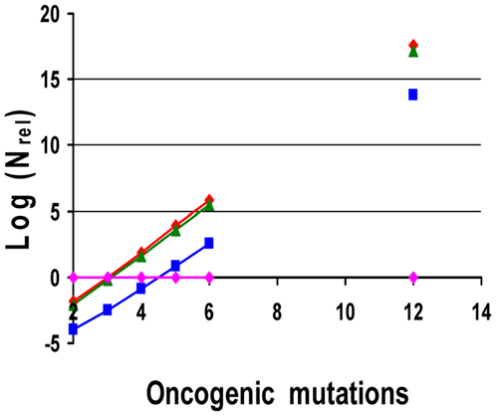
Relative efficiency of mutator compared to non-mutator pathways in the presence of negative clonal selection. The logarithm of N_rel_ (equal to the logarithm of P_rel_), the relative prevalence or efficiency of mutator pathways compared to non-mutator pathways, is plotted as a function of the number of oncogenic mutations required for malignant transformation, for varying values of negative clonal selection. Mutator pathways lead to 50% of clinical cancers when log (N_rel_) = 0 (pink line), and are favored for all positive values of log (N_rel_) (above pink line). The negative clonal selection parameter N_RFLN-D_ (N_reduced fitness loci net-dominant_) is an indicator of the vulnerability of the genome to mutations which may reduce cellular fitness [17]. It consists of the number of loci, in base pairs, single copy mutation of which may reduce fitness of the lineage, where the loci are divided into subclasses, and the number in each subclass is multiplied by the probability that a mutation of it will lead to a fitness reduction as a function of genetic and environmental context. It is varied from 0 (no negative clonal selection, constant fitness, red), to intermediate (N_RFLN-D_ = 9.8×10^4^, green) to high (N_RFLN-D_ = 9.8×10^5^, blue). Low negative clonal selection (N_RFLN-D_ = 9.8×10^3^) is not shown, as it is superimposable on no negative clonal selection for the plotted parameter values. Whereas mutator pathways are favored for 3 or more oncogenic mutations required for transformation at no or intermediate negative clonal selection, under strong negative clonal selection 5 or more oncogenic mutations must be required for transformation before mutator pathways are favored. Calculated using equations [28–30], with the wild type mutation rate k_mut_ = 10^−11^, the fold increase in mutation rate due to a mutator mutation α = 100, the number of cell generations T = 5000, and the number of loci, mutation of which leads to a mutator mutation, N_ML_ = 100.

The relative importance of mutator pathways increases with increasing number of required oncogenic mutations for malignant transformation, but in contrast to the other cases, the minimal number of oncogenic mutations at which mutator pathways are favored [log (N_rel 1:0_)>0] varies depending on the strength of negative clonal selection, as shown in Figure 6 for a fold increase in mutation rate α = 100, a wild type mutation rate k_mut_ = 10^−11^, and a number of cell generations to cancer T = 5,000. At maximal negative clonal selection, there must be at least 5 oncogenic mutations required for malignant transformation before mutator pathways are favored. Additional more detailed results are given in Supplementary Table S3. In general mutator pathways are favored when 5 or more oncogenic mutations are required for malignant transformation and not favored when 2 or fewer oncogenic mutations are required. Results when 3 or 4 oncogenic mutations are required depend on parameter values.

**Figure 7:**
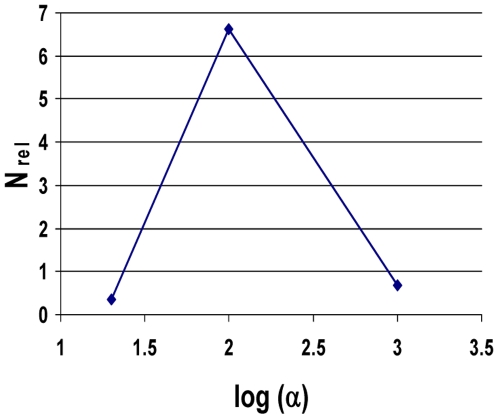
Relative efficiency of mutator pathways with negative clonal selection versus magnitude of mutation rate increase. The relative efficiency of mutator compared to non-mutator pathways N_rel_ is plotted against the logarithm of the fold-increase in mutation rate due to a mutator mutation α, for three α values (20, 100, 1000) at high negative clonal selection (net number of dominant reduced fitness loci N_RFLN-D_ = 9.8×10^5^). In contrast to all models without negative clonal selection, a 100-fold increase in the mutation rate leads to a more efficient mutator pathway than a 1,000-fold increase. Calculated using equations [28–30], with the number of oncogenic mutations required for malignant transformation C = 5; number of cell generations to cancer T = 5000; wild type mutation rate k_mut_ = 10^−11^; number of loci, mutation of which leads to a mutator mutation, N_ML_ = 100.

Whereas in the absence of negative clonal selection, higher values of the fold increase α in mutation rate due to a mutator mutation, number of cell generations T, and wild type mutation rate k_mut_ generally favor mutator mutations, in the presence of negative clonal selection the relative importance of mutator pathways depends on the parameter values in a complex way. Figure 7 depicts the relative prevalence of mutator pathways with initial mutator mutations N_rel 1∶0_ as a function of α, for the highest levels of negative clonal selection, with the number of oncogenic mutations required for cancer C = 5, the number of cell generations to cancer T = 5,000, and the wild type mutation rate k_mut_ = 10^−11^, illustrating the decrease in the relative importance of mutator pathways beyond an optimum. Supplementary Table S3 shows the relative probability of a mutator pathway with an initial mutator mutation compared to no mutator pathway, in the presence of negative clonal selection (N_rel 1∶0, NCS_) for numerous combinations of parameter values not shown in the Figures.

## Discussion

This paper highlights a new approach to evaluation of the importance of somatic mutator mutations in tumorigenesis. The relative efficiencies of mutator and non-mutator pathways are considered, thus circumventing the need for comparisons of absolute rates of tumorigenesis with epidemiologic data, with their inherent limitations such as the assumption that every malignant cell becomes a clinical cancer. It was previously shown that mutator pathways are generally more efficient in the setting of constant fitness despite requiring an additional step for acquisition of the mutator mutation [15]. More efficient mechanisms are likely to play a proportionately larger role in tumorigenesis.

However, tumorigenesis involves changes in fitness including decreased fitness leading to extinction (negative clonal selection) and increased fitness leading to lineage selection and expansion. These could have profound effects on the relative importance of mutator pathways in tumorigenesis. Hence, in the present work I quantitatively consider the variations in fitness that have historically been raised as counterarguments to the mutator hypothesis. This analysis confirms the importance of mutator pathways in tumorigenesis across most representative fitness landscapes. Importantly, the results do not negate the important role for simultaneous lineage expansion and selection.

In all cases, mutator pathways are favored when there are more oncogenic mutations required for cancer. Depending on the situation, mutator pathways will be favored when the number of required oncogenic mutations exceeds the range of 3–5. Genome wide sequencing of solid tumors suggests tumors may harbor between 14–20 oncogenic mutations [26]–[27].

The functional dependence of the importance of mutators on other key parameters varies depending on the different cases considered. For example, in some cases, increasing the number of cell generations T enhances the importance of mutators, in others it has no effect, and in still others there is an optimum beyond which further increases in T decrease the importance of mutators. The case which best matches clinical phenomena needs to be determined and may vary for different cancer types. For constant fitness models, negative clonal selection models, and incremental lineage expansion models, mutator mutations are more efficient if they occur early. However, for cooperative lineage expansion models, whether it is more efficient for the mutator mutations to occur before (case 2) or after (case 3) lineage expansion onset depends on the parameters, and can be determined in any particular circumstance by comparing N_rel_, the relative efficiency of mutator compared to non-mutator pathways, for case 2 and case 3.

While the models generally predict that mutator mutations are an early event, this may be difficult to verify experimentally. Mutator mutations may occur in only a minority of premalignant lesions, but the mutator premalignant lesions are predicted to overwhelmingly be the ones that actually develop into cancers. The model allows the calculation of the fraction of pre-malignant lesions with a given number of oncogenic mutations that harbor mutator mutations in exactly the same way that it allows calculation of the fraction of malignant tumors which develop by the mutator pathway as a function of the number of oncogenic mutations. It therefore follows that premalignant lesions with two or less oncogenic mutations will be less likely to harbor a mutator mutation, but that those few mutator early premalignant lesions will ultimately produce the majority of advanced premalignant lesions and cancers. Since the mutator lesions become progressively enriched with increasing progress towards malignancy, any experimental technique which looks at average mutation frequency across many premalignant lesions might falsely conclude that a mutator mutation is a “late event”. One would need to be able to look at the presence or absence of a mutator mutation in large numbers of premalignant lesions and/or cells individually to verify this prediction. The mutator lesions and/or cells may remain in the minority until lesions with 3–4 or more oncogenic mutations are surveyed.

The analysis predicts that for genetic DNA repair syndromes such as xeroderma pigmentosum (XP) and hereditary non-polyposis colon cancer (HNPCC), simple relationships will obtain between the observed degree of increased risk, the number of oncogenic mutations required for malignant transformation, the time to cancer onset, and the increased mutation rate as a result of the disorder. These parameters are all measurable, with the exception of the number of oncogenic mutations required for malignant transformation, which can be estimated based on evolving molecular knowledge in experimental systems.

The model also predicts that in syndromes with inherited mutator mutations such as HNPCC, premalignant lesions such as polyps will be more efficiently converted to cancer. Because of this very efficient acquisition of additional oncogenic mutations, HNPCC can result in a higher number of cancers without a higher number of polyps. The greater enhancement of number of cancers compared to the enhancement of the number of polyps can be anticipated based on the greater number of oncogenic mutations required to produce the former. In contrast, in a genetic cancer syndrome which is not based on a mutator mutation, familial adenomatous polyposis (FAP), we see an increase in the number of polyps at least equivalent to the increase in the number of cancers.

The model predicts, again correctly, that the mutator mutation accessible through HNPCC will result in an increased risk of colon cancer but not of embryonal carcinomas. Thus, in HNPCC, in which a single further mutation confers a mutator phenotype, there is no increased risk of embryonal cancers, which require fewer oncogenic mutations in their formation, despite an increased colon cancer risk corresponding to the larger number of oncogenic mutations in the pathogenesis of colon cancer. In retinoblastoma, few oncogenic mutations are required, but as the mutator mutation is also the pathognomonic oncogenic mutation, its occurrence is favored.

In the case of negative clonal selection, tumorigenic efficiency does not continue to increase with increased mutation rate, and actually decreases beyond an optimum rate. The optimal mutation rate for tumor evolution is consistently higher than one published estimate of the mutation rate of embryonic stem cells [23], and is generally higher than the estimates for mutation rates of somatic cells [24]. If we assume the estimate of the stem cell mutation rate is correct, and that it has evolved to be optimal for species evolution, the comparison emphasizes that while tumor and species evolution may be partially analogous, they may differ in important quantitative and even qualitative features. Thus, mutator mutations may be important for the evolution of tumors without being important in species evolution.

The optimum mutation rate for tumor evolution estimated by this treatment is analogous to the optimum mutation rate for viruses under selection pressure from antiviral therapy [28]–[29], which is the reciprocal of the viral genome length. The present work differs in that it assumes multiple steps are required for malignant transformation (hence the factor C), that not all sites when mutated lead to reduced cellular fitness (hence N_RFLN-D_ rather than the full genome length), that either member of a diploid gene pair can suffer a reduced fitness mutation (hence the factor of 2), and that multiple cell generations, rather than 1, need to be considered (hence the factor T).

The increased optimal mutation rate calculated here for tumors is consistent with the several experimental demonstrations of increased tumor incidence in mice genetically engineered to have mutator mutations [30]–[32], as well as the several hundred fold higher random mutation frequency in human tumors observed experimentally compared to surrounding normal tissues, when mutations are measured at a genetically neutral site [20]. Note these mutations in human tumors may be present in only a very small minority of cells within a tumor mass, and can be detected only by PCR amplification from single copies [20]. While the observed random mutation frequency also depends on the number of cell generations, it is unlikely the cell generation number increase in tumors is several hundred fold relative to normal tissues. In colon cancer for example, the normal tissue arises from a highly proliferative stem cell compartment which has been proliferating since birth. Finally, the derivation of an optimal mutation rate greater than wild type is consistent with studies of bacteria competing for survival in culture, which show the winners have an increased, but not excessively increased, mutation frequency [Loh E, Salk JS, Loeb LA, unpublished].

I will now briefly consider the more speculative questions raised in the introduction.

1. As mutator pathways appear to predominate in most instances, can the diversity and complexity of tumors be addressed by current therapeutic strategies?
  Clinically, mutator lineages will lead to enhanced heterogeneity within tumors, enhancing the probability that a sub-population of tumor cells manifests pre-existing resistance to therapy. In addition, sensitive cells within mutator lineages will evolve to resistance more quickly. Both pre-existing and acquired resistance emphasize the need for therapeutic combinations [22].
2. Can tumor diversity and genetic instability be used to stratify patients for prognosis and therapy?
  The question of how many agents to give in combination, especially when limited by toxicity, may depend on measures of underlying tumor diversity and plasticity.
3. Can therapy be designed to increase the mutation rate in tumors beyond the optimum derived in this paper, resulting in lethal mutagenesis?
  The proposed anti-cancer strategy termed “lethal mutagenesis” [33] involves increasing the mutation rate to the point where negative clonal selection threatens survival of the malignant lineage. The mathematical model of negative clonal selection can potentially be adapted to allow estimation of the tumor mutation rates which need to be reached to achieve this effect, as well as the mutational “therapeutic window” between tumor and normal tissue. Current inhibitors of cell cycle checkpoint kinases, under preclinical and clinical development as chemotherapy and radiation sensitizers, exemplify methods to further enhance the mutagenic effect of therapy, by bypassing pauses for repair of DNA damage [34]. These agents may be selective based on the absence of a functional p53 checkpoint in tumors.
4. Can the onset of tumors be delayed to beyond the human lifetime, and therefore prevented, by small decreases in the mutation rate?
  As cancer is generally a disease of the elderly, only a modest delay in its onset is required to reduce its importance compared to other causes of mortality. As the efficiency of tumorigenesis is a function of the mutation rate raised to the power of the number of oncogenic mutations required for malignant transformation, only a very modest reduction in mutation rate would be required to delay the onset of cancer [35]. This in turn suggests that prevention of cancer through public health and/or pharmacologic measures aimed at reducing the mutation rate could be effective.
5. What are the underlying reasons for quantitative differences between tumor and species evolution?
  The genome may be more tolerant of mutation in the context of tumorigenesis, in which homeostasis need not be maintained in the whole organism, than in the evolution of species, in which this homeostatic constraint must be obeyed. This may result in a higher optimum mutation rate for tumor evolution.

In summary, this paper provides a general quantitative evaluation of the relative importance of mutator pathways compared to non-mutator pathways in tumorigenesis, accounting for fitness variation and selection, thus directly addressing the major historical criticisms of the mutator hypothesis. Mutator pathways predominate in most but not all instances. The optimal mutation rate is higher for tumor evolution than for species evolution.

## Methods

The present work builds on methods previously published concerning both tumor evolution at constant fitness [15] and negative clonal selection in the absence of tumor evolution [17]. However, it significantly extends this work by providing a general analytic solution for determining the relative efficiency of mutator pathways, compared to non-mutator pathways, for fitness pathways involving increased fitness of malignant lineages and their precursors, as well as providing an analysis of negative clonal selection in the presence of tumor evolution.

All the models are “multi-hit models”, in that malignant lineages, with C oncogenic mutations, arise by mutation from precursor lineages with C−1 mutations, which in turn arise from precursor lineages with C−2 mutations, etc. In the constant fitness case, the probability of having C oncogenic mutations at time T is derived for the non-mutator pathway based on minimal assumptions. The corresponding quantity for the mutator pathway is then the probability weighted integral of all the possible pathways with an additional somatic mutator mutation occurring at any time between 0 and T [15].

In the original negative clonal selection work, the number of viable lineages surviving decays exponentially with a time constant β equal to the product of the mutation rate per base per cell generation, k_mut_, and a general indicator of the vulnerability of cellular fitness to dominant mutation, N_RFLN-D_, the net number of loci in bases, single copy mutation of which will reduce fitness, adjusted for the probability of fitness reduction associated with each locus, summed over possible genetic and environmental contexts. The asssumption that all lineages with reduced fitness will become extinct is nearly true for large populations, and dominant reduced fitness mutations are of greater quantitative significance than recessive ones [17].

The models do not assume that different lineages are competing for an ecologic niche of a fixed size, as is often the case in evolutionary game theory and related techniques [36]. It is assumed that wild type lineages maintain their numbers and the size of their ecological niche, lineages with reduced fitness become extinct, and lineages with increased fitness, including malignant lineages and their precursors, increase exponentially at rates determined by their relative fitness, potentially breaking anatomic barriers to form malignant or benign tumors respectively within expanded niches. These assumptions, intuitively aligned with the occurrence of benign premalignant lesions in solid tumors, enable the derivation of complete analytic solutions incorporating both multi-hit tumorigenesis and arbitrarily varied fitness landscapes.

The exponential growth rates are determined by relative fitness R. This follows from the assumption that the change in relative numbers of a lineage per unit time is proportional to its relative fitness, i.e.: 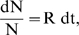
(2)where N is the number of cells, dN/N is the change in their relative number, R is the relative fitness, and dt is an instant of time measured in cell generations. Integrating [2] from 0 to T cell generations leads to an exponential growth equation depending on relative fitness, i.e. 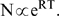
(3)


Exponential growth is thought to characterize nascent tumors until they reach a limiting size [37]. Note a relative fitness of zero corresponds to wild type fitness and results in constant numbers over time.

The lineage expansion models (cases 1–3) are formulated in terms of “expectation values,” or the mean number of malignant cell lineages generated by a particular tumorigenic mechanism at a reference timepoint. This is in contrast to the constant fitness model in which a single cell lineage experiences a limited number of cell divisions, so that mutation in any single nucleotide locus during the lifetime of a single cell and its progeny is a rare event. Probabilities of rare events joined as “or” approximately add (P(A or B)≈P(A)+P(B)), and the constant fitness model is expressed in terms of probabilities [15]. In the presence of lineage expansion, a large population of N cells may result from the lineage of a single cell, where N is equal to or greater than the reciprocal of the per nucleotide mutation rate per cell generation times the number of cell generations [38]. In that instance, the probability that at least one cell in this population harbors a mutation at a particular nucleotide locus may approach 1, and probabilities of individual events joined as “or” do not simply add. However, expectation values are still additive under an “or” operation, simplifying the theoretical treatment. Biologically, this corresponds to the postulate that not every malignant lineage leads to a clinical cancer, and the mechanisms which produce the most malignant lineages are most likely to contribute to tumorigenesis.

The models are designed for large cell populations. In scenarios where there are small clusters of cells, such as intestinal crypts, the models may be thought of as reflecting the average behavior of a population of crypts, including the fact that benign tumors or polyps may arise in some.

Other key assumptions, parameters, and parameter values have been previously reviewed [15], [17]. Key input and output parameters and their values are also given in the “Results” section and in Table 1.

Below we give the equations used to calculate the results in this paper and the general strategy for deriving them. Full derivations are available upon request. These full derivations also include demonstrations that all cases are identical to the previously derived constant fitness case [15] in the limit where the natural log fitness advantage R (cases 1–3) or susceptibility constant β for negative clonal selection (case 4) approach 0, serving as a check on the mathematics.

### Case 1: Incremental Lineage Expansion

Given the assumption that any fully malignant lineage with C oncogenic mutations has an equal chance of becoming a clinical cancer, N_rel_, the relative number of clinical cancers due to mutator compared to non-mutator pathways at time T or earlier, is given by: 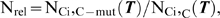
(4)where N_Ci,C-mut_ ***(T)*** and N_Ci,C_ ***(T)*** are the number of new lineages initiated with C oncogenic mutations, and C oncogenic mutations are required for malignant transformation, up to and including time T, for mutator and non-mutator pathways, respectively.

The model is a multi-hit model wherein C oncogenic mutations are required for tumorigenesis, and is described by a network of ordinary differential and integral equations. Lineages with no oncogenic mutations do not increase their numbers (denoted N_0_), but lineages with n oncogenic mutations increase in number by a factor of e^nR/C^ each *wild type* cell generation. Hyperproliferative mutations which decrease the generation time are appropriately factored into the value of R.

Lineages with n mutations also increase their numbers by initiation events: i.e., mutations from the lineages with n−1 oncogenic mutations. The rate of initiation of new lineages with n oncogenic mutations at any instant t, dN_ni,C_ ***(t)***, is given by the product of the number of cells with n−1 oncogenic mutations at that instant, N_n−1,C_ ***(t)***; the mutation rate, k_mut_; the number of remaining unmutated oncogene loci, N_OL_-n+1 (where N_OL_ is the number of oncogenic loci available for mutation in a wild type cell); and a factor to account for more cell generations per unit time if there is a hyper-proliferative mutation, e^Rp^: 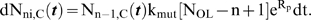
(5)


The negligible effect of mutation in decreasing precursor populations is ignored, simplifying the treatment. The total number of lineages with n mutations initiated by time T is the integral of the instantaneous initiation rate from 0 to T: 
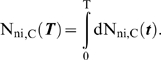
(6)


The number of cells in a given class of lineage by time T is given by the integral of the instantaneous initiation rate at time t, multiplied by the lineage expansion from time t to T for that lineage class, over the interval t = [0,T].



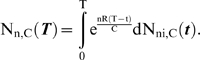
(7)


In particular, malignant initiation events occur from lineages with C−1 oncogenic mutations, as they acquire their Cth oncogenic mutation: 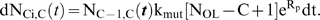
(8)


The total number of malignant initiation events by time T is simply the integral of this instantaneous initiation rate from 0 to T: 
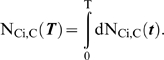
(9)


In summary, the instantaneous initiation rate of lineages with one oncogenic mutation is given by a first order differential equation. Based on the instantaneous initiation rate of lineages with one oncogenic mutation, and their lineage expansion, one can derive an expression for the number of cells with one oncogenic mutation as a function of time. The instantaneous rate of initiation of lineages with two oncogenic mutations is proportional to the number of cells with one oncogenic mutation at any given time. In turn, based on the instantaneous initiation rate of lineages with two oncogenic mutations, and their lineage expansion, we derive an expression for the number of cells with two oncogenic mutations at any point in time. The instantaneous initiation rate of cells with 3 oncogenic mutations is then proportional to the number of cells with 2 oncogenic mutations, and so on.

Expressions for the number of cells and lineage initiations were derived for several values of n (number of oncogenic mutations) and C (number of oncogenic mutations required for malignant transformation), and based on the algebraic details, general expressions were hypothesized for all n and C, verified for the cases explicitly derived, and proven for all n and C by mathematical induction.

For mutator pathways, the mutator mutation is assumed to occur first, based on previous work suggesting that mutator pathways are more efficient if the mutator mutation occurs early [15]. The rate of formation of cells with the original mutator mutation, dN_0,C-mut_ ***(t)***, is given by the product of the initial number of cells N_0_, the number of mutator loci N_ML_, and the mutation rate: 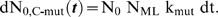
(10)


Once a lineage with a mutator mutation is formed, analysis of its progress parallels that of the non-mutator pathway, except for the increase in the mutation rate constant by the factor α.

The numbers of malignant cell initiations by both mutator and non-mutator pathways is the sum of exponentials representing lineages with 0 to C−1 oncogenic mutations. α_50%_, the minimum fold increase in mutation rate at which mutator pathways account for 50% of observed cancers (derived by setting N_rel_ = 1), is approximated by considering only the most rapidly growing exponential: 
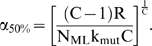
(11)


The exact expression, considering all exponentials, is: 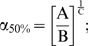
(12a)

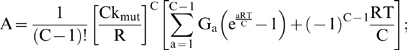
(12b)

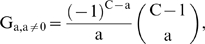
(12c) where 
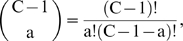
(12d)the number of combinations, or ways “a” oncogenes can be selected from a set of C−1 oncogenes, without regard to the order of selection; 
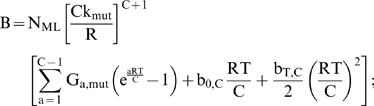
(12e)

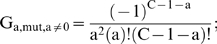
(12f)

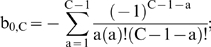
(12g)

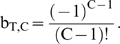
(12h)


A and B are proportional to the number of initiation events by time T for non-mutator and mutator pathways (with α = 1), respectively. The approximate expression [11] is within 1% of the exact expressions [12a–h] for the vast majority of cases, and within 5% for all cases examined.

N_rel_, the relative efficiency of mutator compared to non-mutator pathways, is given by the following expression when the mutator mutations increase the mutation rate by a factor of α: 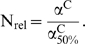
(13)


### Case 2: Cooperative Lineage Expansion, Early Mutator Mutation

In this case, there is no change in fitness until D≤C oncogenic mutations have occurred. These first D steps, with or without a mutator mutation, can therefore be analyzed using the strategy previously outlined for the constant fitness case [15]. In this case, for the mutator pathway, the mutator mutation is allowed to occur anywhere up to (but not including) the point where D oncogenic mutations have occurred, and these D possible time intervals are summed.

Once D oncogenic mutations have occurred, the lineage has a natural log fitness advantage R, and expands by a factor of e^R^ per *wild type* cell generation (see equations [2–3] above). During this period, the fitness is also constant, although greater than it was prior to the D oncogenic mutations. The total number of cells with C−1 oncogenic mutations (by non-mutator or mutator pathways) at time T is given by the integral over t from 0 to T of the product of: the number of cells N_D−1,C_ ***(t)*** or N_D−1,C-mut_ ***(t)*** with D−1 oncogenic mutations (without or with a mutator mutation, respectively) at time t; the instantaneous mutation rate per locus for conversion to cells with D oncogenic mutations (k_mut_ for non-mutators, α k_mut_ for mutators); the number of unmutated oncogenic loci at the time of conversion to cells with D oncogenic loci (N_OL_−D+1), the exponential lineage expansion of cells with D oncogenic mutations from time t to time T (e^R(T-t)^); and the probability that any progeny of this expanded lineage would have acquired the final C−D−1 oncogenic mutations in time T−t (P_C−1|D,C_ ***(T−t)*** or P_C-1|D,C-mut_ ***(T−t)***) for non-mutators and mutators, respectively, adjusted for the absence or presence of mutator mutations and the increased number of cell generations per unit time, if the fitness increase includes an increase in proliferation rate: 
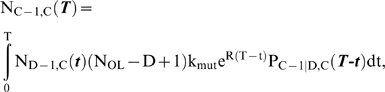
(14a)

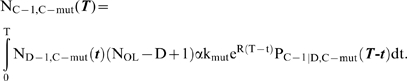
(14b)


The probability of the expanded lineage acquiring C−D−1 oncogenic mutations in time T-t is the product of the number of ways of selecting C−D−1 oncogenic mutations from N_OL_-D remaining unmutated oncogenic loci, 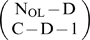; and the single step mutation probability per locus (k_mut_(T−t) for non-mutators, α k_mut_(T−t) for mutators, adjusted by a factor of 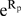 to account for increased number of cell generations per unit time if there is a hyperproliferative mutation), raised to the (C−D−1)st power: 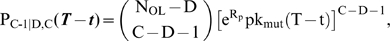
(15a)

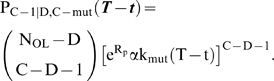
(15b)


As in case 1, we use the number of cells with C−1 oncogenic mutations to calculate the instantaneous rate of malignant initiation events at any time, integrating that from 0 to T to obtain the number of malignant initiation events at or prior to T. N_rel_, the relative number of clinical cancers due to mutator compared to non-mutator pathways at time T or earlier, is given as before by the ratio of total number of malignant initiation events at or before time T for mutator divided by non-mutator pathways (see equation [4]). α_50%_ is again derived by setting N_rel_ = 1.

An approximate expression for α_50%_, in the limit of significant lineage expansion, and increasingly accurate as the fitness advantage R and the fold mutation rate increase α get larger, is: 
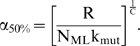
(16) N_rel_ is again given by [13].

### Case 3: Cooperative Lineage Expansion, Late Mutator Mutation

In this case, there is no change in fitness and no mutator mutation until D≤C oncogenic mutations have occurred. These first D steps can therefore be analyzed using the strategy previously outlined for the non-mutator pathway in the constant fitness case [15].

Once D oncogenic mutations have occurred, the lineage has a natural log fitness advantage R, and expands by a factor of e^R^ per *wild type* cell generation. During this time, an additional C−D oncogenic mutations will also occur, with a mutator mutation occurring anywhere from the 1^st^ to (C−D)th step in the process. During this period, the fitness is also constant, although greater than it was prior to the D oncogenic mutations.

The total number of cells with C−1 oncogenic mutations at time T by a mutator mechanism, N_C−1,C-mut_ ***(T)***, is given by the sum, over the possible steps k at which a mutator mutation can occur, of double integrals. These double integrals from t equals 0 to T are of the product of the number of cells with D−1 oncogenic mutations at time t, N_D−1,C-mut_ ***(t)***; the instantaneous rate of conversion of these cells to cells with D oncogenic mutations, (N_OL_−D+1) k_mut_dt; the lineage expansion from time t to T, e^R(T−t)^; and an internal integral representing the likelihood of subsequent acquisition of the remaining C−D−1 oncogenic mutations and a mutator mutation in time T-t. This internal integral is over t' equals 0 to T-t, and the integrand is the product of the probability of having k−1 additional oncogenic mutations between time t and time t+t' before the mutator mutation occurs, P_k−1,t'_; the instantaneous rate of occurrence of the mutator mutation (adjusted to account for the reduced cell generation time in the presence of a hyperproliferative mutation) at time t', e^Rp^ N_ML_ k_mut_ dt'; and the probability that the remaining C−D−k oncogenic mutations will occur in the remaining T−t−t' cell generations, P_C−1,k−1,t',t_.



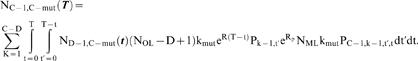
(17)


In analogy with previous arguments, 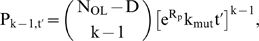
(18)where the first term represents the number of possible combinations of k−1 oncogenic mutations from N_OL_-D unmutated oncogenes, 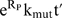 is the probability of one oncogenic locus being mutated in the time t' (given that any hyperproliferative mutation increases the mutation rate per *wild type* cell generation by a factor 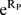), and 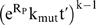 is the probability of k−1 oncogenic loci being independently mutated in this time, and 
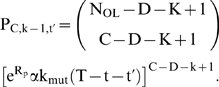
(19)


The number of cells with C−1 oncogenic mutations at time T by non-mutator pathways is the same as in case 2.

As in cases 1 and 2, we use the number of cells with C−1 oncogenic mutations to calculate the instantaneous rate of malignant initiation events at any time, integrating that from 0 to T to obtain the number of initiation events at or prior to T. N_rel_, the relative number of clinical cancers due to mutator compared to non-mutator pathways at time T or earlier, is again given by the ratio of total number of malignant initiation events at or before time T for mutator divided by non-mutator pathways (see equation [4]). α_50%_ is again derived by setting N_rel_ = 1.

An approximate expression for α_50%_, in the limit of significant lineage expansion, and increasingly accurate as the fitness advantage R and the fold mutation rate increase α get larger, is: 
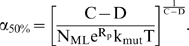
(20) N_rel_ is given by: 
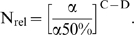
(21)


In the case of cooperative lineage expansion, we can determine whether an early or late mutator mutation is more efficient by comparing the respective values of N_rel_. A late mutator (case 3) is more efficient (and therefore more likely) than an early mutator (case 2) if and only if: 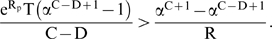
(22)


For α≫1, [22] is well approximated by the simple expression:
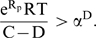
(23)


We see that early mutator mutations (i.e. before the fitness increase) are much more likely in the cooperative case for larger values of D.

### Case 4: Negative Clonal Selection

In this case, lineages are subject to negative clonal selection (NCS), or random dominant reduced fitness (RF) mutations that are deleterious with a certain probability proportional to N_RFLN-D_. Lineages with reduced fitness become extinct. The loss of fitness is described by a first order differential equation, leading to exponential decay of surviving lineages (P_S_ is the probability of survival), with exponent given by minus the product of the susceptibility constant β, the number 2 (given diploid cells), α (for mutator lineages only) and the number of cell generations T. In turn, β is the product of the mutation rate constant k_mut_ and the net number of dominant RF loci

N_RFLN-D_ [17]:
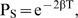
(24a)

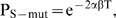
(24b)

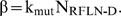
(24c)


As this case does not involve lineage expansion, the model can be expressed in terms of probabilities rather than expectation values.

The probability of a malignant lineage initiation by a non-mutator pathway by time T, P_cancer, 0, NCS_, is the product of the probability of surviving negative clonal selection for T cell generations (equation [24a]) and the probability of having C oncogenic mutations at time T [15]:
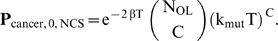
(25)


The maximum probability of malignancy as a function of underlying mutation rate can be found by differentiating this expression with respect to the mutation rate k_mut_ (bearing in mind equation [24c] for β), and setting this derivative equal to zero, leading to equation [1] from the “Results” section. The optimal mutation rate for carcinogenesis calculated in this way is generally significantly higher than the wild type mutation rate. The derivation is analogous to that derived from quasispecies theory [28]–[29], except it considers the need to acquire C mutations rather than 1, T cell generations rather than 1, N_RFLN-D_ rather than the full genome length as the size of the target which can mutate to reduced fitness, and the factor of 2 to account for a diploid genome.

The mutator pathway probability of carcinogenesis is evaluated assuming the mutator mutation occurs first. Loss of lineages due to NCS is more rapid after a mutator mutation, but so is the acquisition of oncogenic mutations. The probability of malignant lineage initiation with a mutator mutation as step 1, P_cancer, 1, NCS_, is the integral from t equals 0 to T of the product of the instantaneous rate of occurrence of the mutator mutation at time t (N_ML_ k_mut_ dt); the probability of surviving negative clonal selection until time t without a mutator mutation, P_0, t, NCS_; and the probability of acquiring C oncogenic mutations while surviving negative clonal selection in time T–t after enhancement of mutation rate by an mutator mutation, P_C-mut, 0, t, NCS_: 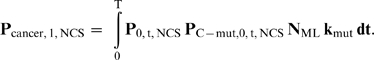
(26)


The probability of surviving negative clonal selection until time t, P_0, t, NCS_, is given by [24a] and [24c] with T = t. The probability of acquiring C oncogenic mutations while surviving negative clonal selection in time T–t after enhancement of mutation rate by a mutator mutation, P_C-mut, 0, t, NCS_, is given by the product of: the probability of surviving negative clonal selection for T-t cell generations given a mutator mutation, e^−2αβ(T−t)^; the number of ways to choose C oncogenes from a set of N_OL_ oncogenes, 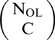; and the probability of C oncogenic mutations occuring as independent events in time T−t, [αk_mut_(T−t)]^C^:
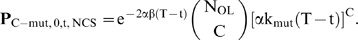
(27)


The relative efficiency or probability (N_rel_ or P_rel_) of a mutator pathway with an initial mutator mutation to that of a non-mutator pathway in the presence of negative clonal selection is the ratio of the malignant initiation probabilities for mutator vs. non-mutator pathways, and is given by:
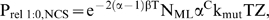
(28) where 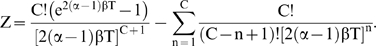
(29)


For (α−1)βT≪1, an alternative expression for Z must be used to maintain adequate computational precision: 
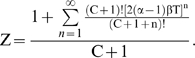
(30)


### Lemmas Required to Reproduce the Derivations in Cases 1–3

To reproduce the derivations above, the following identities involving factorials are required. Proofs of these identities are available on request.



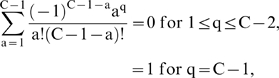
(31)

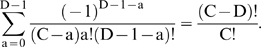
(32)


## Supporting Information

Table S1 α_50%_: incremental and cooperative lineage expansion cases (cases 1–3). α_50%_, the minimum fold increase in mutation rate at which mutator pathways account for 50% of observed cancers; C, the number of oncogenic mutations required for commitment to cancer; D, the number of oncogenic mutations required for fitness increase in the cooperative lineage expansion model; e^R^, the relative fitness advantage per cell generation of malignant lineages compared to wild type; R_P_, the component of R due to enhanced proliferation rate; k_mut_, the wild type mutation rate per nucleotide per cell generation; T, the number of wild type cell generations at which the efficiency comparison is made (lineages with hyperproliferative mutations may have undergone more generations); LE, lineage expansion; MM, mutator mutation. For the cooperative lineage expansion case, α_50%_ is determined by the smaller of the two values (early and late mutator mutation). For the cooperative lineage expansion case with late mutator mutation, D and R_P_ are required for the calculation, and we assume in these examples D = 2 and R_P_≈0.5 R. Calculated using equations [11], [12a–h], [16], and [20] of the main paper, or as described for the constant fitness model (reference 15, main paper), assuming N_ML_, the number of “mutator loci” in nucleotides, mutation of which may lead to genetic instability, is 100. The fraction of cancers arising with an initial mutator mutation causing fold mutation increase α in their pathogenesis is given by α^C^/(α^C^+α_50%_^C^).
(0.11 MB DOC)

Table S2 α_50%_ for high fitness advantage R (R = 2): incremental and cooperative lineage expansion cases (cases 1–3). α_50%_, the minimum fold increase in mutation rate at which mutator pathways account for 50% of observed cancers; C, the number of oncogenic mutations required for commitment to cancer; D, the number of oncogenic mutations required for fitness increase in the cooperative lineage expansion model; e^R^, the relative fitness advantage per cell generation of malignant lineages compared to wild type; R_P_, the component of R due to enhanced proliferation rate; k_mut_, the wild type mutation rate per nucleotide per cell generation; T, the number of wild type cell generations at which the efficiency comparison is made (lineages with hyperproliferative mutations may have undergone more generations); LE, lineage expansion; MM, mutator mutation. For the cooperative lineage expansion case, α_50%_ is determined by the smaller of the two values (early and late mutator mutation). For the cooperative lineage expansion case with late mutator mutation, D and R_P_ are required for the calculation, and we assume in these examples D = 2 except for when C = 2 (in which case we assume D = 1) and R_P_ = 1.31 (the maximum contribution to R from enhancing cell survival is 0.69, equivalent to doubling cell numbers each generation, and the remainder of R must therefore come from an increased proliferation rate). Calculated using [11–12a–h], [16], and [20] of the main paper, and as described (reference 15 of the main paper) for the constant fitness case, assuming N_ML_, the number of “mutator loci” in nucleotides, mutation of which may lead to genetic instability, is 100. The fraction of cancers arising with an initial mutator mutation causing fold mutation increase α in their pathogenesis is given by α^C^/(α^C^+α_50%_^C^).
(0.10 MB DOC)

Table S3 P_rel 1∶0, NCS_, Relative Efficiency of Mutator Pathways During Negative Clonal Selection (Case 4). P_rel 1∶0, NCS_, the ratio of cancers arising with and without an initial mutator mutation in their pathogenesis in the presence of negative clonal selection (NCS); C, the number of oncogenic mutations required for commitment to cancer; N_RFLN-D_, the net number of dominant reduced fitness loci; α, the fold increase in mutation rate due to a mutator mutation; T, the number of wild type cell generations at which the efficiency comparison is made (lineages with hyperproliferative mutations may have undergone more generations). Calculated using equations [28]–[30] of the main paper, assuming N_ML_, the number of “mutator loci” in nucleotides, mutation of which may lead to genetic instability, is 100, and k_mut_, the wild type mutation rate per base per cell generation, is 10^−11^. The fraction of cancers arising with an initial mutator mutation in their pathogenesis is given by P_rel 1∶0, NCS_/(1+P_rel 1∶0, NCS_).
(0.07 MB DOC)
