# Supplementary material for: Mutator Mutations Enhance Tumorigenic Efficiency across Fitness Landscapes
Source: PLoS One. 2009 Jun 10;4(6):e5860. doi: 10.1371/journal.pone.0005860 (PMC2690659; doi:10.1371/journal.pone.0005860)
Supplement: Table S1 — α50%: incremental and cooperative lineage expansion cases (cases 1–3). α50%, the minimum fold increase in mutation rate at which mutator pathways account for 50% of observed cancers; C, the number of oncogenic mutations required for commitment to cancer; D, the number of oncogenic mutations required for fitness increase in the cooperative lineage expansion model; eR, the relative fitness advantage per cell generation of malignant lineages compared to wild type; RP, the component of R due to enhanced proliferation rate; kmut, the wild type mutation rate per nucleotide per cell generation; T, the number of wild type cell generations at which the efficiency comparison is made (lineages with hyperproliferative mutations may have undergone more generations); LE, lineage expansion; MM, mutator mutation. For the cooperative lineage expansion case, α50% is determined by the smaller of the two values (early and late mutator mutation). For the cooperative lineage expansion case with late mutator mutation, D and RP are required for the calculation, and we assume in these examples D = 2 and RP≈0.5 R. Calculated using equations [11], [12a–h], [16], and [20] of the main paper, or as described for the constant fitness model (reference 15, main paper), assuming NML, the number of “mutator loci” in nucleotides, mutation of which may lead to genetic instability, is 100. The fraction of cancers arising with an initial mutator mutation causing fold mutation increase α in their pathogenesis is given by αC/(αC+α50% C). (0.11 MB DOC) [file pone.0005860.s001.doc]

**Supplementary Table S1. α_50%_: incremental and cooperative lineage expansion cases (cases 1-3)**.

| C | D | e^R^ | e^RP^ | k**_mut_** | T | Approx. α**_50%_,**  LE,  Incremental | Exact α**_50%_**  LE,  Incremental | α**_50%_**  LE,  Cooperative,  Early MM | α**_50%_**  LE,  Cooperative,  Late MM | α**_50%_**  Constant fitness |
| --- | --- | --- | --- | --- | --- | --- | --- | --- | --- | --- |
| 2 | 1 | 2 | 1.4 | 10**^-11^** | 170 | 18600 | 18600 | 26300 | 4.15X10**^6^** | 4200 |
| 2 | 1 | 2 | 1.4 | 10**^-11^** | 5000 | 18600 | 18600 | 26300 | 1.41X10**^5^** | 775 |
| 2 | 1 | 2 | 1.4 | 10**^-9^** | 170 | 1860 | 1860 | 2630 | 4.15X10**^4^** | 420 |
| 2 | 1 | 2 | 1.4 | 10**^-9^** | 5000 | 1860 | 1860 | 2630 | 1410 | 77 |
| 2 | 1 | 1.2 | 1.1 | 10**^-11^** | 170 | 10000 | 10000 | 14100 | 5.30X10**^6^** | 4200 |
| 2 | 1 | 1.2 | 1.1 | 10**^-11^** | 5000 | 10000 | 10000 | 14100 | 1.81X10**^5^** | 775 |
| 2 | 1 | 1.2 | 1.1 | 10**^-9^** | 170 | 1000 | 1000 | 1410 | 5.30X10**^4^** | 420 |
| 2 | 1 | 1.2 | 1.1 | 10**^-9^** | 5000 | 1000 | 1000 | 1410 | 1810 | 77 |
| 3 | 2 | 2 | 1.4 | 10**^-11^** | 170 | 770 | 770 | 880 | 4.2 X 10**^6^** | 290 |
| 3 | 2 | 2 | 1.4 | 10**^-11^** | 5000 | 770 | 770 | 880 | 1.4 X 10**^5^** | 93 |
| 3 | 2 | 2 | 1.4 | 10**^-9^** | 170 | 170 | 170 | 190 | 42000 | 62 |
| 3 | 2 | 2 | 1.4 | 10**^-9^** | 5000 | 170 | 170 | 190 | 1400 | 20 |
| 3 | 2 | 1.2 | 1.1 | 10**^-11^** | 170 | 510 | 510 | 590 | 5.30X10**^6^** | 290 |
| 3 | 2 | 1.2 | 1.1 | 10**^-11^** | 5000 | 510 | 510 | 590 | 1.81X10**^5^** | 93 |
| 3 | 2 | 1.2 | 1.1 | 10**^-9^** | 170 | 110 | 110 | 130 | 53000 | 62 |
| 3 | 2 | 1.2 | 1.1 | 10**^-9^** | 5000 | 110 | 110 | 130 | 1800 | 20 |
| 4 | 2 | 2 | 1.4 | 10**^-11^** | 170 | 151 | 151 | 162 | 2880 | 74 |
| 4 | 2 | 2 | 1.4 | 10**^-11^** | 5000 | 151 | 151 | 162 | 531 | 32 |
| 4 | 2 | 2 | 1.4 | 10**^-9^** | 170 | 48 | 48 | 51 | 288 | 23 |
| 4 | 2 | 2 | 1.4 | 10**^-9^** | 5000 | 48 | 48 | 51 | 53 | 10 |
| 4 | 2 | 1.2 | 1.1 | 10**^-11^** | 170 | 111 | 111 | 119 | 3270 | 74 |
| 4 | 2 | 1.2 | 1.1 | 10**^-11^** | 5000 | 111 | 111 | 119 | 602 | 32 |
| 4 | 2 | 1.2 | 1.1 | 10**^-9^** | 170 | 35 | 35 | 38 | 327 | 23 |
| 4 | 2 | 1.2 | 1.1 | 10**^-9^** | 5000 | 35 | 35 | 38 | 60 | 10 |
| 5 | 2 | 2 | 1.4 | 10**^-11^** | 170 | 56 | 56 | 59 | 232 | 32 |
| 5 | 2 | 2 | 1.4 | 10**^-11^** | 5000 | 56 | 56 | 59 | 75 | 16 |
| 5 | 2 | 2 | 1.4 | 10**^-9^** | 170 | 22 | 22 | 23 | 50 | 13 |
| 5 | 2 | 2 | 1.4 | 10**^-9^** | 5000 | 22 | 22 | 23 | 16 | 6.5 |
| 5 | 2 | 1.2 | 1.1 | 10**^-11^** | 170 | 44 | 44 | 45 | 252 | 32 |
| 5 | 2 | 1.2 | 1.1 | 10**^-11^** | 5000 | 44 | 44 | 45 | 82 | 16 |
| 5 | 2 | 1.2 | 1.1 | 10**^-9^** | 170 | 17 | 17 | 18 | 55 | 13 |
| 5 | 2 | 1.2 | 1.1 | 10**^-9^** | 5000 | 17 | 17 | 18 | 18 | 6.5 |
| 6 | 2 | 2 | 1.4 | 10**^-11^** | 170 | 29 | 29 | 30 | 63 | 19 |
| 6 | 2 | 2 | 1.4 | 10**^-11^** | 5000 | 29 | 29 | 30 | 27 | 11 |
| 6 | 2 | 2 | 1.4 | 10**^-9^** | 170 | 13 | 13 | 14 | 20 | 8.6 |
| 6 | 2 | 2 | 1.4 | 10**^-9^** | 5000 | 13 | 13 | 14 | 8.7 | 4.9 |
| 6 | 2 | 1.2 | 1.1 | 10**^-11^** | 170 | 23 | 23 | 24 | 68 | 19 |
| 6 | 2 | 1.2 | 1.1 | 10**^-11^** | 5000 | 23 | 23 | 24 | 29 | 11 |
| 6 | 2 | 1.2 | 1.1 | 10**^-9^** | 170 | 11 | 11 | 11 | 22 | 8.6 |
| 6 | 2 | 1.2 | 1.1 | 10**^-9^** | 5000 | 11 | 11 | 11 | 9.3 | 4.9 |
| 12 | 2 | 2 | 1.4 | 10**^-11^** | 170 | 5.4 | 5.4 | 5.5 | 5.7 | 4.5 |
| 12 | 2 | 2 | 1.4 | 10**^-11^** | 5000 | 5.4 | 5.4 | 5.5 | 4.2 | 3.4 |
| 12 | 2 | 2 | 1.4 | 10**^-9^** | 170 | 3.7 | 3.7 | 3.7 | 3.7 | 3.1 |
| 12 | 2 | 2 | 1.4 | 10**^-9^** | 5000 | 3.7 | 3.7 | 3.7 | 2.6 | 2.3 |
| 12 | 2 | 1.2 | 1.1 | 10**^-11^** | 170 | 4.9 | 4.7 | 4.9 | 5.9 | 4.5 |
| 12 | 2 | 1.2 | 1.1 | 10**^-11^** | 5000 | 4.9 | 4.9 | 4.9 | 4.3 | 3.4 |
| 12 | 2 | 1.2 | 1.1 | 10**^-9^** | 170 | 3.3 | 3.2 | 3.4 | 3.8 | 3.1 |
| 12 | 2 | 1.2 | 1.1 | 10**^-9^** | 5000 | 3.3 | 3.3 | 3.4 | 2.7 | 2.3 |

α_50%_, the minimum fold increase in mutation rate at which mutator pathways account for 50% of observed cancers; C, the number of oncogenic mutations required for commitment to cancer; D, the number of oncogenic mutations required for fitness increase in the cooperative lineage expansion model; e^R^, the relative fitness advantage per cell generation of malignant lineages compared to wild type; R_P_, the component of R due to enhanced proliferation rate; k_mut_, the wild type mutation rate per nucleotide per cell generation; T, the number of wild type cell generations at which the efficiency comparison is made (lineages with hyperproliferative mutations may have undergone more generations); LE, lineage expansion; MM, mutator mutation. For the cooperative lineage expansion case, α_50%_ is determined by the smaller of the two values (early and late mutator mutation). For the cooperative lineage expansion case with late mutator mutation, D and R_P_ are required for the calculation, and we assume in these examples D=2 (except when C =2, D=1) and R_P_ ≈ 0.5 R. Calculated using equations [11]-[12a-h], [16], and [20] of the main paper, or as described for the constant fitness model (reference 15 , main paper), assuming N_ML_, the number of “mutator loci” in nucleotides, mutation of which may lead to genetic instability, is 100. The fraction of cancers arising with an initial mutator mutation causing fold mutation increase α in their pathogenesis is given by αC/ (α^C^ + α_50%_^C^).
