# Supplementary material for: Mutator Mutations Enhance Tumorigenic Efficiency across Fitness Landscapes
Source: PLoS One. 2009 Jun 10;4(6):e5860. doi: 10.1371/journal.pone.0005860 (PMC2690659; doi:10.1371/journal.pone.0005860)
Supplement: Table S3 — Prel 1∶0, NCS, Relative Efficiency of Mutator Pathways During Negative Clonal Selection (Case 4). Prel 1∶0, NCS, the ratio of cancers arising with and without an initial mutator mutation in their pathogenesis in the presence of negative clonal selection (NCS); C, the number of oncogenic mutations required for commitment to cancer; NRFLN-D, the net number of dominant reduced fitness loci; α, the fold increase in mutation rate due to a mutator mutation; T, the number of wild type cell generations at which the efficiency comparison is made (lineages with hyperproliferative mutations may have undergone more generations). Calculated using equations [28]–[30] of the main paper, assuming NML, the number of “mutator loci” in nucleotides, mutation of which may lead to genetic instability, is 100, and kmut, the wild type mutation rate per base per cell generation, is 10−11. The fraction of cancers arising with an initial mutator mutation in their pathogenesis is given by Prel 1∶0, NCS/(1+Prel 1∶0, NCS). (0.07 MB DOC) [file pone.0005860.s003.doc]

**Supplementary Table S3. P_rel 1:0, NCS_, Relative Efficiency of Mutator Pathways During Negative Clonal Selection (Case 4).**

| C | T | α | No NCS | N_RFLN-D_ = 9.8 X 10^3^ | N_RFLN-D_ = 9.8 X 10^4^ | N_RFLN-D_ = 9.8 X 10^5^ |
| --- | --- | --- | --- | --- | --- | --- |
| 2 | 5000 | 1000 | 1.7 | 8.2 X 10^-1^ | 1.1 X 10^-2^ | 1.1 X 10^-5^ |
| 2 | 170 | 5000 | 1.4 | 1.3 | 4.3 X 10^-1^ | 1.8 X 10^-3^ |
| 3 | 170 | 1000 | 4.3 X 10^1^ | 4.1 X 10^1^ | 3.3 X 10^1^ | 3.5 |
| 3 | 5000 | 20 | 1.0 X 10^-2^ | 9.9 X 10^-3^ | 8.6 X 10^-3^ | 2.4 X 10^-3^ |
| 3 | 5000 | 100 | 1.3 | 1.2 | 5.8 X 10^-1^ | 3.3 X 10^-3^ |
| 3 | 5000 | 1000 | 1.3 X 10^3^ | 5.8 X 10^2^ | 3.2 | 3.3 X 10^-4^ |
| 4 | 170 | 100 | 3.4 | 3.4 | 3.3 | 2.6 |
| 4 | 170 | 1000 | 3.4 X 10^4^ | 3.3 X 10^4^ | 2.6 X 10^4^ | 2.4 X 10^3^ |
| 4 | 5000 | 20 | 1.6 X 10^-1^ | 1.6 X 10^-1^ | 1.4 X 10^-1^ | 3.5 X 10^-2^ |
| 4 | 5000 | 100 | 1.0 X 10^2^ | 9.2 X 10^1^ | 4.5 X 10^1^ | 1.3 X 10^-1^ |
| 4 | 5000 | 1000 | 1.0 X 10^6^ | 4.5 X 10^5^ | 1.3 X 10^3^ | 1.3 X 10^-2^ |
| 5 | 170 | 20 | 9.1 X 10^-2^ | 9.1 X 10^-2^ | 9.0 X 10^-2^ | 8.6 X 10^-2^ |
| 5 | 170 | 100 | 2.8 X 10^2^ | 2.8 X 10^2^ | 2.8 X 10^2^ | 2.1 X 10^2^ |
| 5 | 5000 | 20 | 2.7 | 2.6 | 2.3 | 5.6 X 10^-1^ |
| 5 | 5000 | 100 | 8.3 X 10^3^ | 7.7 X 10^3^ | 3.7 X 10^3^ | 6.6 |
| 5 | 5000 | 1000 | 8.3 X 10^8^ | 3.6 X 10^8^ | 6.3 X 10^5^ | 6.8 X 10^-1^ |
| 6 | 170 | 20 | 1.6 | 1.6 | 1.5 | 1.5 |
| 6 | 170 | 100 | 2.4 X 10^4^ | 2.4 X 10^4^ | 2.4 X 10^4^ | 1.8 X 10^4^ |
| 6 | 5000 | 10 | 7.1 X 10^-1^ | 7.1 X 10^-1^ | 6.6 X 10^-1^ | 3.3 X 10^-1^ |
| 6 | 5000 | 20 | 4.6 X 10^1^ | 4.6 X 10^1^ | 3.9 X 10^1^ | 9.2 |
| 6 | 5000 | 100 | 7.1 X 10^5^ | 6.8 X 10^5^ | 3.1 X 10^5^ | 3.8 X 10^2^ |

P_rel 1:0, NCS_, the ratio of cancers arising with and without an initial mutator mutation in their pathogenesis in the presence of negative clonal selection (NCS); C, the number of oncogenic mutations required for commitment to cancer; N_RFLN-D_, the net number of dominant reduced fitness loci; α, the fold increase in mutation rate due to a mutator mutation; T, the number of wild type cell generations at which the efficiency comparison is made (lineages with hyperproliferative mutations may have undergone more generations). Calculated using equations [28-30] of the main paper, assuming N_ML_, the number of “mutator loci” in nucleotides, mutation of which may lead to genetic instability, is 100, and k_mut_, the wild type mutation rate per base per cell generation, is 10^-11^. The fraction of cancers arising with an initial mutator mutation in their pathogenesis is given by P_rel 1:0, NCS_ /(1 + P_rel 1:0, NCS_).
